# Research Progress on the Immunogenicity and Regeneration of Acellular Adipose Matrix: A Mini Review

**DOI:** 10.3389/fbioe.2022.881523

**Published:** 2022-06-06

**Authors:** Kaiyang Liu, Yunfan He, Feng Lu

**Affiliations:** Department of Plastic and Cosmetic Surgery, Nanfang Hospital, Southern Medical University, Guangzhou, China

**Keywords:** acellular adipose matrix, transplantation, immunogenicity, inflammatory cells, regeneration

## Abstract

Acellular adipose matrix (AAM) has received increasing attention for soft tissue reconstruction, due to its abundant source, high long-term retention rate and *in vivo* adipogenic induction ability. However, the current decellularization methods inevitably affect native extracellular matrix (ECM) properties, and the residual antigens can trigger adverse immune reactions after transplantation. The behavior of host inflammatory cells mainly decides the regeneration of AAM after transplantation. In this review, recent knowledge of inflammatory cells for acellular matrix regeneration will be discussed. These advancements will inform further development of AAM products with better properties.

## Introduction

Soft tissue defect remains a challenge for esthetic and reconstructive surgery. Trauma, congenital anomalies or iatrogenic causes can lead to this defect, which affects psychological functioning and patient’s quality of life. Plastic surgeons typically utilize autologous tissue flap transfer for reconstruction of soft tissue defects (Vaienti et al., 2005; Zhang et al., 2016). However, the tenuous nature of microsurgical tissue transfer and donor-site morbidity increases the difficulty of surgical operation, which requires an adequately trained surgeon. Fat grafting is demonstrated to be effective and safe for the treatment of small- and mid-sized volume deficiencies. However, clinicians continue to face challenges such as donor site morbidity, graft loss, calcification and oil cysts (Nguyen et al., 1990; Coleman, 2006; Khouri et al., 2012; Kølle et al., 2013). Hyaluronic acid, collagen and polymethyl methacrylate are all common implantable biomaterials. These biomaterials have the advantages of easy access and no injury to the donor area over autologous fat transplantation. However, they have the problems of foreign body reaction and inflammation, distortion and repeated injections due to biomaterial absorption (Alam et al., 2008; Spector and Lim, 2016; Van Nieuwenhove et al., 2017).

The extracellular matrix (ECM) is a cell-secreted three-dimensional structure that provides mechanical and structural supports to the tissues, which can also serve as a reservoir and a place for active ion, nutrient, water, metabolite and signal exchange (Gattazzo et al., 2014; Mendibil et al., 2020). Therefore, ECM acts as the microenvironment in which tissue-resident cells attach, communicate and interact, thereby regulating cell dynamics and behavior as well as maintaining tissue-specific functions and phenotypes. Similarly, decellularized ECM scaffold allows the seeding and proliferation of specific cells, while being degraded by the host tissue and replaced with new tissue (Massaro et al., 2021). Acellular adipose matrix (AAM) has received increasing attention for reconstruction of soft tissue defects, due to its abundant source, high long-term retention rate and *in vivo* adipogenic induction ability. The processing techniques for AAM generally include the removal of cellular components and lipids, while maintaining the basic ECM architecture and biological activity. Collagen type I, collagen type IV and laminin are the most essential ECM proteins in adipose tissues (Mendibil et al., 2020), (Costa et al., 2017). In addition, adipogenesis can be induced by extracellular proteins such as fibroblast growth factor, insulin-like growth factor, transforming growth factor beta and bone morphogenetic proteins in the AAM (Kayabolen et al. 2017).

Immune reaction can be triggered when the biomaterials are introduced to the human body (Cravedi et al., 2017). Although the use of decellularized biomaterials for regenerative medicine and tissue engineering reduces transplantation-related risks (Crapo et al., 2011; Sart et al., 2020; Peña et al., 2021), the current decellularization approaches are not able to completely eliminate all immunogenic antigens (Crapo et al., 2011), (Brown et al., 2011; Kawecki et al., 2018). Typically, the immune reaction consists of adaptive and innate responses (Hillebrandt et al., 2019). The innate response is dependent on the body’s ability to recognize potentially harmful pathogens and rapidly recruit immune cells to diminish them through inflammatory reactions. The adaptive response is manifested by antigen-specific reactions. This is the case of allo/xenograft rejection caused by the dysregulation of major histocompatibility complex (MHC), which is the most serious risk associated with transplantation (Massaro et al., 2021), (Wong and Griffiths, 2014). According to our previous study, immunocamouflage MHC by methoxy polyethylene glycol could effectively improve the regeneration properties of xenogeneic AAM (Liu et al., 2021). On the other hand, orderly infiltration and transformation of host inflammatory cells are highly essential for adipose tissue regeneration in fat transplantation (Cai et al., 2017a; Cai et al., 2017b; Cai et al., 2018; Liu et al., 2018). Appropriate infiltration of inflammatory cells (e.g., neutrophils and monocytes) at the early stage of transplantation and timely polarization of macrophages from M1 to M2 could promote adipogenic and retention rates in xenogeneic AAM (Liu et al., 2021). Therefore, the reaction of host immune system to AAM can determine the regeneration outcome. In this review, we highlight recent advancements in inflammatory cell behavior and their interaction with AAM after transplantation.

### Immunogenicity of Acellular Adipose Matrix

Generally, immunogenicity is the ability of a substance or molecule to induce a specific immune response (Mahanty et al., 2015). Incomplete decellularization is the main source of immunogenicity in AAM (Crapo et al., 2011; Keane et al., 2012). The expression of MHC antigens is intrinsically linked to immunogenicity. MHC is composed of a cluster of genes that can determine whether the cells, tissues or biological products are accepted or rejected. DNA fragments are the most common residual cellular materials that have been associated with AAM immunogenicity (Massaro et al., 2021). Although a certain amount of DNA (<50 ng/mg AAM dry weight with a fragment length of <200 bp) is considered acceptable (Dalgliesh et al., 2018; Chakraborty et al., 2020; Massaro et al., 2021), there are no definitive data on the actual effect of DNA residue on the degradation and remodeling of AAM grafts. The alpha-Gal (α-Gal; Gal1-3Gal1-4GlcNAc-R) epitope persisting on the cytomembrane is another essential factor in xenogeneic AAM rejection (Chen et al., 1999; Naso et al., 2011). This epitope is expressed on the surface of most mammalian tissues, except for humans and primates. In humans, the xenograft with α-Gal expression triggers the activation of specific antibodies, thus resulting in xenograft rejection (Stahl et al., 2018). Although AAM products have entered the stage of clinical trials and shown their safety (Kokai et al., 2019; Gold et al., 2020; Kokai et al., 2020; Anderson et al., 2022), the actual remaining antigens in AAM are unclear and *in vivo* immunological studies are still very limited. At least 92% lipid antigen removal is the threshold level of residual antigenicity necessary to overcome recipient graft-specific adaptive humoral immune response in rabbit transplant model (Dalgliesh et al., 2018). However, the exact data of α-Gal, MHC and DNA residues in the products under this threshold are still unclear.

After decellularization or proteolytic digestion processes, alterations in the structures of functional proteins, glycoproteins and glycosaminoglycans in the native ECM are unavoidable. The ECM protein, such as hyaluronan, collagen types I and IV, versican, aggrecan, laminin and α1β1 integrin, are still integrated with the ECM after decellularization or proteolytic digestion processes (Dziki et al., 2017; Chakraborty et al., 2020). These motifs help maintain the secondary immunity, B cell differentiation, antibody production, and chemokine receptor (CXCR)-1 and 2-regulated neutrophil attraction, (Partington et al., 2013; Youngstrom et al., 2013). Meanwhile, ECM components such as hyaluronan fragments, tenascins, and sulfated proteoglycans following decellularization or proteolytic digestion processes could amplify inflammation (Gaudet and Popovich, 2014; Dziki et al., 2018). However, some ECM damage may be tolerated by the innate immune response, provided that macromolecular structure is maintained (Dalgliesh et al., 2018).

Despite the above-mentioned potential immunogenicity, an immune response is less likely to be provoked in the AAM (Massaro et al., 2021). Considering the genetic similarities among species, the presence of xenogeneic proteins can be revealed if they are functionally homologous to their human counterparts. Xenogeneic ECM proteins can effectively interact with human binding receptors, especially in decellularized scaffolds, through binding to peptide motifs or matrix bound proteins that are highly conserved across multiple species (Massaro et al., 2021).

### From the Very Beginning After Transplantation

The host response following biomaterial implantation can be classified as three phases: protein adsorption, inflammation, and foreign body reaction. The spontaneous adsorption of blood components, such as sugars, lipids and proteins, onto the graft surface occurs within seconds after implantation (Wilson et al., 2005; Mariani et al., 2019). Simultaneously, at the biomaterial interface, the surrounding tissue forms a provisional matrix (e.g., seroma, blood clot and thrombus) that releases bioactive agents (e.g., PDGF, TGF-β, CXCL4 and LTB4) to govern the subsequent phases of inflammation (Anderson et al., 2008; Franz et al., 2011; Brown et al., 2012; Bryers et al., 2012; Anderson, 2015). Changes in the conformation and composition of the adsorbed proteins trigger inflammatory responses and a series of processes including the recruitment and adhesion of innate immune cells (e.g., monocytes and neutrophils) (Anderson, 2015; Chung et al., 2017; Selders et al., 2017; Zhou and Groth, 2018). During the inflammation phase, acute inflammation is predominantly determined by the appearance of neutrophils that enter the implant sites through damaged vessels (Anderson et al., 2008; Zhou and Groth, 2018). Later, the chronic inflammatory response at the graft is initiated by mononuclear cells (e.g., lymphocytes and monocytes), which are recruited by the signals released by the activated and apoptotic neutrophils (Anderson et al., 2008). More importantly, the activation of an immune response cascade has been proven to be a positive phenomenon because inflammation is a physiological process after transplantation (Kawecki et al., 2018). Therefore, early post-transplant inflammatory response plays a vital role in regulating AAM regeneration.

### Neutrophils-Mediated Immune Reaction and Implant Remodeling

**FIGURE 1:**
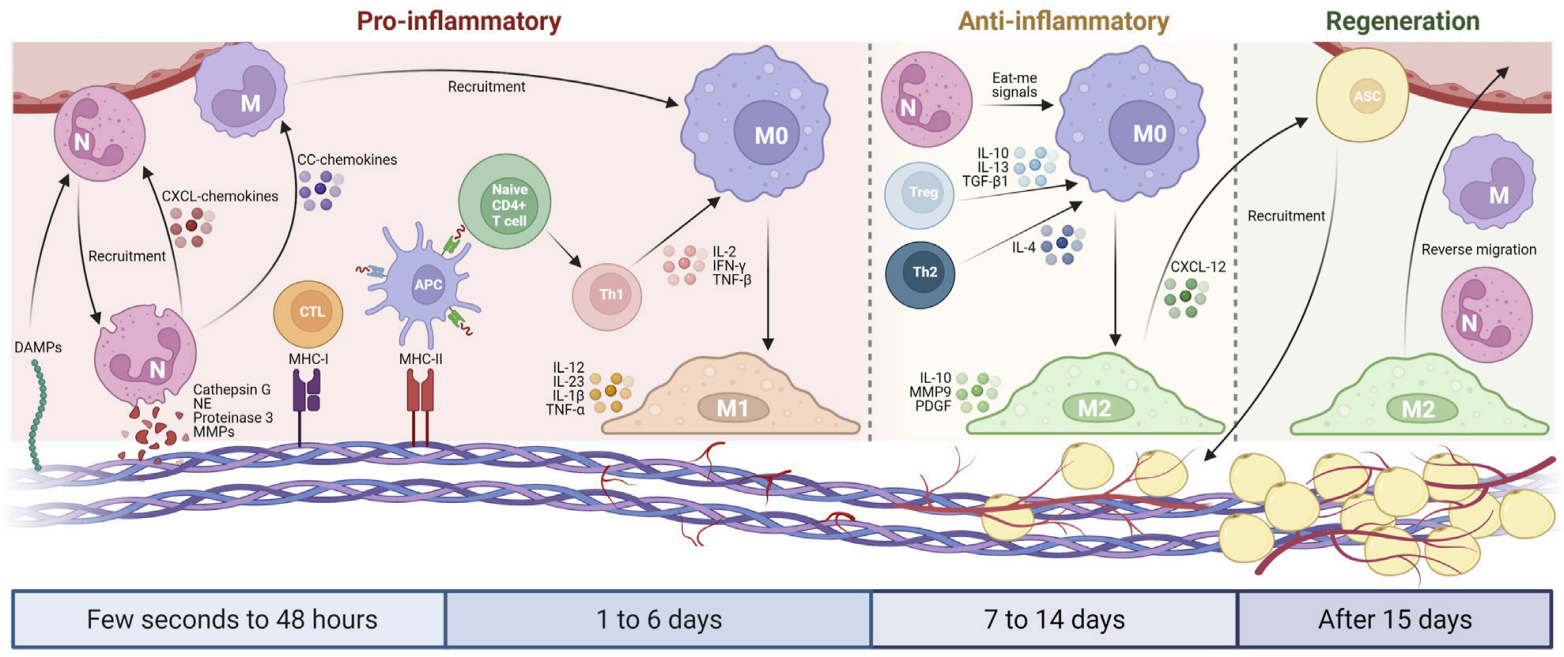
Schematic diagram of the host inflammatory cell implantation in AAM. From a few seconds to 48 h, DAMPs cause the initial recruitment of neutrophils, which secrete serine proteases and MMPs to cleave AAM components. Neutrophils produce CXCL-chemokines and CC-chemokines, which recruit more neutrophils and monocytes from the circulation. The remaining MHC molecules in AAM activate lymphocytes that promote M1 macrophage polarization via pro-inflammatory cytokines during 1–6 days after implantation. From day 7–14, the host blood vessels begin to grow in the AAM, thereby inducing adipogenesis. Neutrophils, Treg cells, and Th2 lymphocytes subsequently promote M2 macrophage polarization that translates pro-inflammatory into an anti-inflammatory environment. After 15 days, the inflammation level of AAM gradually decreased, the neovascularization further developed, and the adipogenesis process continued with the participation of M2 macrophages. Created with BioRender.com.

Neutrophils are the first cells that appear at the implant sites, and they play a critical role during the early graft reaction. During the leukocyte adhesion cascade process, neutrophils are recruited from the bloodstream to a site of injury via chemotaxis (Ley et al., 2007; Rosales, 2018). After adhesion to the graft surface, they are activated for matrix reprogramming, angiogenesis and regeneration (López-Boado et al., 2004; Dupré-Crochet et al., 2013). Within 24–48 h, neutrophils infiltrate the graft and generate peptides, enzymes and neutrophil extracellular traps, which subsequently recruit immune cells (e.g., monocytes and lymphocytes) and affect surrounding tissue (Anderson et al., 2008; Björnsdottir et al., 2015; Perobelli et al., 2015). Afterward, they will undergo apoptosis, and subsequently being engulfed, phagocytozed and digested by the attracted macrophages (Figure 1).

Neutrophils migrate to the graft in three phases: forward migration, recruitment amplification, and reverse migration (de Oliveira et al., 2016). Damage-associated molecular patterns (DAMPs) are recognized by specific pattern recognition receptors, which causes the initial recruitment of neutrophils (de Oliveira et al., 2016). DAMPs include N-formyl peptides, ECM components and DNA proteins, all of which can serve as the products of tissue destruction during the decellularization process. CXCL8 family of chemokines (CXCL8 chemokines) can attract more distant neutrophils (de Oliveira et al., 2016). Following transmigration, the recruitment of neutrophils can be amplified via CXCL8 chemokines and leukotriene B4 (Zhang et al., 2015). The third phase is characterized by the removal of neutrophils from the graft via macrophage phagocytosis, apoptosis or reverse migration.

Angiogenesis is a tightly coupled spatiotemporal process that requires cells to degrade and remodel the surrounding ECM (Hanjaya-Putra et al., 2012; Zhang et al., 2015; Crosby and Zoldan, 2019). Firstly, the basement membrane is degraded to allow the capillary sprout to grow in an existing blood vessel (Crosby and Zoldan, 2019). This is followed by further ECM degradation that allows the sprouting endothelial cells to invade the host tissue, thus creating a zone for the sprouting vascular lumen (Crosby and Zoldan, 2019). Human neutrophils express a specific set of neutrophil serine proteases, namely, proteinase 3, neutrophil elastase (NE) and cathepsin G (Owen and Campbell, 1995; Kessenbrock et al., 2011). These three serine proteases can cleave ECM components such as collagen, fibronectin, elastin, proteoglycans and laminin (Pipoly and Crouch, 1987; Rao et al., 1991; Owen and Campbell, 1995). Neutrophils also produce matrix metalloproteinases (MMPs) that can induce tissue regeneration, angiogenesis and matrix remodeling (Selders et al., 2017). MMPs can function as collagenases and gelatinases to break down connective tissues. However, in an appropriate amount, MMPs can contribute to tissue remodeling, angiogenesis and matrix reprogramming, which are consistent with the early resolution of immune-mediated responses (Mantovani et al., 2011).

Neutrophils also release CC chemokines to promote monocyte chemotaxis (Selders et al., 2017), and regulate the activation and recruitment of dendritic cells and natural killer cells (Kumar and Sharma, 2010; Mantovani et al., 2011; Brinkmann and Zychlinsky, 2012; Chistiakov et al., 2015). In addition, when the neutrophils give off eat-me signals, a specific phenotype is triggered in the engulfing macrophages, and the macrophages polarize into the M2 phenotype to activate a regenerative pathway, thereby negatively modulating inflammatory response (Mantovani et al., 2011). These functions are particularly important for AAM, as the M2-like macrophages can facilitate resolution and matrix remodeling, which in turn improve graft integration. The M2 macrophage polarization transition characterizes the resolution of inflammation, which is additionally triggered by the presence of apoptotic neutrophils (Boni et al., 2019). In contrast, a sustainable number of neutrophils may increase the M1 macrophage recruitment, which induces the fusion of these cells into foreign body giant cells and ultimately results in persistent inflammation (Boni et al., 2019).

Acute inflammation is a normal and essential reaction of the innate immune system, which can lead to a persistent inflammatory response that accelerates biomaterial degradation and tissue damage (Selders et al., 2017). Considering the above-mentioned functions of neutrophils, it is necessary to develop an AAM that can result in limited self-activation of neutrophils to maintain their biodegradability at an appropriate level. However, the exact role of neutrophils in AAM grafts is still unknown, and awaits further investigation.

### Macrophages-Mediated Immune Reaction and Implants Remodeling

Monocyte-derived macrophage plays a critical role in regulating inflammatory responses during the implantation of biomaterials. It determines whether the graft becomes encapsulated, causes persistent inflammation or is completely integrated into the body, thus allowing for tissue regeneration. The two phenotypes of macrophages, M1 and M2, are responsible for cell-biomaterial interaction (Figure 1). Generally, M1 macrophages are involved in the pro-inflammatory response that leads to the development of chronic inflammation, whereas M2 macrophages promote a regenerative response in the tissue that is favourable for successful transplantation (Mirmalek-Sani et al., 2013; Wong and Griffiths, 2014). The fate of the AAM is closely dependent on the monocyte-derived macrophages that exist in the graft (Huleihel et al., 2017).

Within 1–6 days following transplantation, M1 macrophage initiates vascularization in the graft via pro-inflammatory signaling, which involves the upregulated levels of IL-1β, IL-6, IL-12, IL-23 and TNF-α (Brown et al., 2009). Later, M2 subtype can inhibit the fibrous or granuloma encapsulation by releasing IL-10 with rapid iron transport for positive tissue remodeling (Chakraborty et al., 2020). M2 macrophages directly contribute to adipogenesis in adipogenic induction models by promoting angiogenesis and adipogenesis (Han et al., 2015; Li et al., 2016). M2 macrophages also promote stem cell recruitment, preadipocyte survival and vessel remodeling during adipose tissue regeneration by releasing platelet-derived growth factor, MMP-9 and chemokine ligand 12 (Cai et al., 2018; Spiller et al., 2014).

The phenotype and function of macrophages can be altered by physicochemical stimuli, including biomaterial surface topography and roughness (McWhorter et al., 2015; Zhou and Groth, 2018). The fundamental mechanism by which an AAM maintains a dynamic balance between M1 and M2 macrophages is currently unknown (Spiller et al., 2015; Swinehart and Badylak, 2016). The timely transformation of M1 into M2 macrophages is conducive to the adipogenesis of the graft in xenogeneic AAM transplantation model (Liu et al., 2021). Optimization of the decellularization process can generate specific ECM peptides in the bio-scaffold, which leads to cell migration and integration with the host tissue.

Previous studies have shown that a successful ECM-derived biomaterial can effectively promote the plasticity of macrophages, thus allowing them to polarize from M1 to M2 within 7–14 days (Brown et al., 2014; Julier et al., 2017). However, the differences in the functional plasticity of macrophages as well as the roles of their intermediate subtypes (M2a-c) in the immune modulation process remain largely unclarified (Qiu et al., 2018; Robb et al., 2018). These specific groups can play potential roles in tissue remodeling, immune regulation and wound healing. Therefore, future studies on infiltrated macrophages in AAM should refine the classification of M2 macrophages, rather than categorizing them as M2 phenotype. The resolution phase of inflammation begins after 15 days of transplantation, while macrophage infiltration and adipogenesis are still visible 12 weeks after transplantation (He et al., 2018; Lin et al., 2019; Liu et al., 2021). Therefore, it is required to further extend the terminal observation time point of the AAM graft in future studies.

### Lymphocytes-Mediated Immune Reaction and Implant Remodeling

In addition to the monocyte-driven macrophages, lymphocytes may emerge at the inflammatory sites after AAM implantation, thereby recognizing the antigenic fragments on the AAM and activating dendritic cells and macrophages (Chakraborty et al., 2020). Lymphocytes are involved in the B and T cell-mediated adaptive responses by interacting with the MHC molecules expressed on the surface of antigen-presenting cells (APCs) and are identified by the receptors on the T cell surface (Bach et al., 1997). T cells can be used to identify the difference between the peptides (e.g., versican, aggrecan, laminin and integrins) of host cells and the peptides on APCs (Figure 1) (Chakraborty et al., 2020).

MHC class I molecules refer to the peptides on the antigen surface that replicate simultaneously and those proteins present in the cytosolic fractions of the cells, which have shown to activate cytotoxic CD8^+^ T cells (Ghosh et al., 2005). MHC class II molecules are restricted to specific cells called APCs, including dendritic cells, macrophages and B cells. The presentation of peptides by MHC class II on APCs can activate CD4^+^ T cells (Wieczorek et al., 2017). Upon activation, cytotoxic CD8^+^ T cells and CD4^+^ Th1 cells migrate to the graft, where they can activate monocyte-driven and resident macrophages to combat the antigenic motifs (Allman et al., 2001).

T-helper 1 (Th1) lymphocytes produce tumor necrosis factor-β, interferon-γ and interleukin-2, resulting in macrophage activation, stimulation of complement-fixing antibody isotypes and differentiation of CD8^+^ cells to a cytotoxic phenotype (Irwin et al., 1989; Chen et al., 1996). T-helper 2 (Th2) lymphocytes generate cytokines, IL-4, -5, -6 and -10, leading to the production of non-complement-fixing antibody isotypes (Pattison and Krensky, 1997; Sadtler et al., 2016). Regulatory T (Treg) cells are immune regulatory cells that play a crucial role in maintaining immune homeostasis (Wang et al., 2008). Generally, Th1 polarization can contribute to proinflammatory responses, while Th2/Treg polarization is involved in transplant acceptance and constructive remodeling response. The decreased level of Th1:Th2 in grafts may be beneficial to the *in vivo* retention and regeneration of acellular matrix (Allman et al., 2001; Bayrak et al., 2010; Melgar-Lesmes et al., 2017). Our recent study has demonstrated that the increased level of Treg cells from methoxy polyethylene glycol-modified AMM is associated with adipogenic induction and M2 polarization (Liu et al., 2021). mPEG-modified antigens (e.g., MHC molecules) often exhibit a reduced affinity for receptors compared to the unmodified antigens (Webster et al., 2007). The strength of the interactions between MHC and T cell receptor (TCR) is closely associated with the fate of T cells (Kim and Williams, 2010; Josefowicz et al., 2012). Low-affinity antigen-TCR engagement can lead to a decrease in intracellular TCR signaling events that enhance the differentiation of naive T cells into Treg cells (Sauer et al., 2008; Turner et al., 2009). In addition, Treg cells could promote M2 macrophage polarization through the secretion of IL-10, IL-13 and TGF-β1 (Liu et al., 2021). The behavior of T cells in AAM play a key role in graft retention and regeneration, but the exact mechanism needs to be explored further.

It is worth noting that the acellular biomaterial’s properties can have an impact on lymphocyte differentiation. As mentioned above, ECM serves as a reservoir of growth factors and cytokines that persist after decellularization. Hence, the detection and quantification of ECM are of immense importance. After decellularization, surface molecules and peptides (e.g., hyaluronan, collagen fragments, versican, aggrecan, laminin and α1β1 integrin) are retained on the scaffolds, which can regulate immune responses (Thomas et al., 2007; Bollyky et al., 2011). However, the mechanism by which decellularized scaffold components mediate the exact host immune responses remains unknown.

## Conclusion

The innate and adaptive immune responses to AAM, as well as the role of inflammatory cell degradation and remodeling, are still poorly understood. Different molecular structures and individual components of the proteins in AAM elicit specific immune responses in the host tissues after transplantation. These responses can modulate host cell functions, such as inflammatory response, cell migration and progenitor cell differentiation, in the AAM under pathological and physiological conditions, which can facilitate tissue regeneration. Therefore, it is necessary to further elucidate the inflammatory process of AAM after transplantation, in order to enhance the regeneration of AAM *in vivo*.
